# The treatment of amphotericin B-resistant *C neoformans* meningitis: A case report and literature review

**DOI:** 10.1097/MD.0000000000043862

**Published:** 2025-08-29

**Authors:** Liang Long, Qingzi Yan, Can Xiao, Xiang Liu, Yi Yan

**Affiliations:** a Clinical Pharmacy, Xiangtan Central Hospital, Xiangtan, Hunan Province, China; b Department of Orthopedics, Xiangtan Central Hospital, Xiangtan, Hunan Province, China.

**Keywords:** amphotericin B resistance, *C neoformans* meningitis, case report, pharmaceutical care

## Abstract

**Rationale:**

Cryptococcal meningitis is caused by *Cryptococcus neoformans* and *Cryptococcus gattii*, predominantly affects immunocompromised host. Resistance to amphotericin B poses therapeutic challenges, especially in immunocompetent individuals, where evidence is scarce.

**Patient concerns:**

This study reports a case of an old immunocompetent male diagnosed with amphotericin B-resistant *C neoformans* meningitis.

**Diagnoses:**

Amphotericin B-resistant *C neoformans* meningitis.

**Interventions:**

Treatment failure occurred with both induction regimens (amphotericin B deoxycholate/fluconazole for 14 days followed by fluconazole/flucytosine for 29 days), then used salvage therapy combining amphotericin B colloidal dispersion (ABCD) in this amphotericin B-resistant *C neoformans* meningitis.

**Outcomes:**

Successful salvage therapy with ABCD/flucytosine was achieved in a case of amphotericin B-resistant *C neoformans* meningitis. However, the patient ultimately succumbed to multidrug-resistant *Klebsiella pneumoniae* meningitis secondary to prolonged dexamethasone use for ABCD infusion reaction prophylaxis, which induced significant immunosuppression.

**Lessons:**

ABCD is a feasible alternative treatment for amphotericin B-resistant *C neoformans* meningitis. During prolonged ABCD therapy, pharmacists must implement pharmaceutical care to ensure medication safety and mitigate adverse effects, thereby preventing treatment discontinuation or treatment failure.

## 1. Introduction

**Figure 1.**
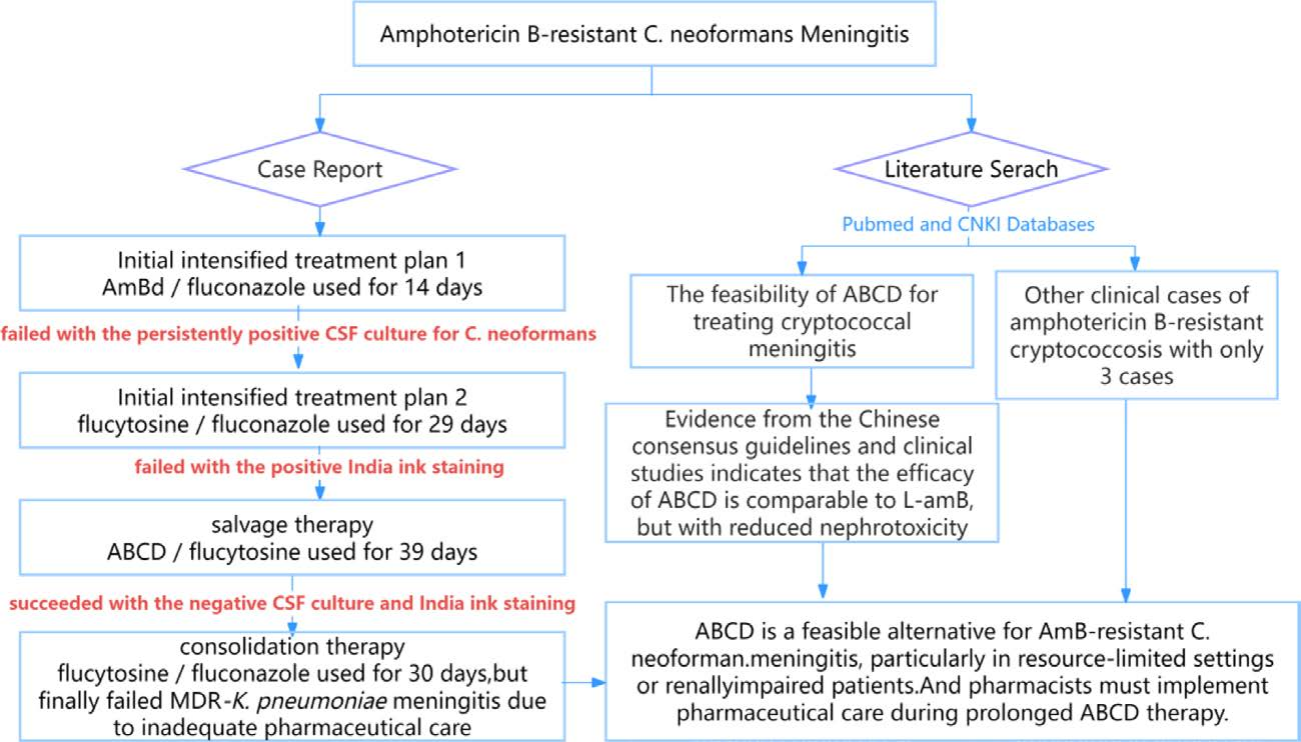
Flow chart of the study.

Cryptococcal meningitis (CM), a central nervous system infection caused by *Cryptococcus* invasion of the meninges or brain parenchyma, is primarily attributed to *Cryptococcus neoformans* and *Cryptococcus gattii*, pathogens associated with high morbidity and mortality.^[1,2]^ While immunocompromised individuals (e.g., those with AIDS, malignancies, or glucocorticoid use) are at an elevated risk, 50% to 70% of Chinese CM patients are immunocompetent.^[3,4]^ Standard CM induction therapy relies on amphotericin B deoxycholate (AmB-D), flucytosine, and triazoles. However, the rising cryptococcal drug resistance, particularly fluconazole resistance, contributes significantly to treatment failure.^[1,5]^ Herein, we report a case of amphotericin B-resistant *C neoformans* meningitis in an immunocompetent host. The treatment algorithm is illustrated in Fig. 1.

## 2. Case report

A 74-year-old immunocompetent male farmer (Han Chinese, 59 kg) was admitted on May 17, 2023, with fever (38.2 °C) and delirium. He had a 3-month history of cognitive decline, initially attributed to cerebral infarction on *magnetic resonance imaging*. Physical examination revealed neck stiffness with otherwise normal vital signs (blood pressure 150/78 mm Hg). The initial diagnoses included fever of unknown origin and poststroke status. Computed tomography revealed mild pulmonary inflammation and lacunar infarction. Despite normal liver and kidney function, concurrent COVID-19 infection prompted ceftriaxone and molnupiravir therapy. By hospital day 6, progressive neurological decline (including visual impairment) led to a CM diagnosis via positive cerebrospinal fluid (CSF) India ink staining and next-generation sequencing confirmation. Treatment timeline: days 1 to 19: AmB-D (0.6 mg/kg/day)/fluconazole (400 mg/day) was failed with the persistently positive CSF culture for *C neoformans* (minimum inhibitory concentration [MIC] = 2 μg/mL); days 20 to 49: flucytosine (100 mg/kg/day)/fluconazole (600 mg/day) was still failed with the positive India ink staining; days 50 to 89: the salvage therapy of amphotericin B colloidal dispersion (ABCD) (4.2 mg/kg/day)/flucytosine (100 mg/kg/day) was succeeded with the negative CSF culture and India ink staining, and dexamethasone 5 mg intramuscular before each ABCD infusion and weight gain (59 → 87 kg) finally; day 90+: the consolidation therapy of flucytosine (100 mg/kg/day)/fluconazole (600 mg/day) kept the clinical stabilization for a long time. The patient had negative CSF cultures and normal hematologic/liver parameters (except elevated creatinine [122 μmol/L] and urea [13.2 μmol/L]). However, the patient developed sudden fever and coma on day 118, despite normal outpatient CSF analysis. The patient died on day 120 due to multidrug-resistant *K* pneumoniae bacterial meningitis. Alterations in the intracranial pressure and CSF test results during antifungal treatment are shown in Table 1. The main treatment drugs for the patient are shown in Table 2.

**Table 1:** Alterations in whole intracranial pressures and CSF tests during antifungal treatment.

Data	Cerebrospinal pressure (mm H_2_O)	CSF tests					
		Protein (mg/L)	Glucose (mmol/L)	Chloride (mmol/L)	India ink staining	WBC (*10^6^/L)	Culture
1st day	180	2051↑	1.39↓	113↓	NA	177↑	*C neoformans*
4th day	250	1940↑	2.21↓	111↓	*Cryptococcus*	171↑	*C neoformans*
6th day	240	1805↑	1.69↓	107↓	*Cryptococcus*	179↑	*C neoformans*
13th day	200	1720↑	1.96↓	122.8	*Cryptococcus*	125↑	*no*
19th day	260	1981↑	1.43↓	105.9↓	*Cryptococcus*	170↑	*C neoformans*
24th day	220	1495↑	2.38↓	107.3↓	*Cryptococcus*	158↑	*C neoformans*
29th day	195	1248↑	2.35↓	114.4↓	NA	139↑	NA
35th day	190	1205↑	2.58	119.1	NA	142↑	NA
46th day	178	989↑	2.51	117.6↓	*Cryptococcus*	152↑	NA
49th day	180	978↑	2.71	116.1	*Cryptococcus*	164↑	NA
64th day	140	540↑	2.89	125.6	NA	16↑	NA
74th day	170	482↑	3.93	126.3	NA	12	NA
104th day	158	462↑	4.36	125.1	NA	10	NA
118th day	160	456	3.75	121.3	NA	11	NA
119th day	220	2055	1.45	96.7	NA	1020	MDR-*K pneumoniae*

Adults’ normal ranges for cerebrospinal pressure, protein, glucose, and chloride are 80 to 180 mm H_2_O at clinical stasis, 150 to 450 mg/L, 2.5 to 4.5 mmol/L, and 118 to 128 mmol/L.

*C neoformans* = *Cryptococcus neoformans*, CSF = cerebrospinal fluid, MDR-*K pneumoniae* = multidrug-resistant *Klebsiella pneumoniae*.

**Table 2:** The main treatment drugs for the patient.

Major drug	Usage and dosage	Start and end date
Molnupiravir	0.8 g po q12h	Days 1–5
Ceftriaxone	2 g ivgtt qd	Days 1–6
Amphotericin B deoxycholate	Increase the dose gradually from 5 mg and keep 35 mg from day 5 ivgtt qd	Days 6–19
Fluconazol	0.4 g ivgtt qd and double the first day	Days 6–19
	0.6 g ivgtt qd	Days 20–49
	0.6 g po qd	Days 90–120
Flucytosine	1.5 g po q6h	Days 20–120
Amphotericin B colloidal dispersion	Increase the dose gradually from 50 mg and keep 250 mg from day 3 ivgtt qd	Days 50–89
Dexamethasone sodium phosphate	5 mg im qd (before amphotericin B colloidal dispersion)	Days 50–89

## 3. Discussion

Cryptococcal resistance is a critical determinant of treatment failure in CM. While fluconazole resistance has been extensively characterized, amphotericin B resistance remains rare and poses significant therapeutic challenges.^[6]^ Through a systematic literature search of the PubMed and CNKI databases using the terms “Cryptococcus,” “drug susceptibility,” and “amphotericin B resistance,” we identified only 3 documented cases of amphotericin B-resistant cryptococcosis: HIV-associated CM: successful salvage therapy with fluconazole monotherapy^[7]^; immunocompetent systemic cryptococcosis: cured with voriconazole^[8]^; triple-therapy failure: AmB-D (1 mg/kg/day) + flucytosine (100 mg/kg/day) + fluconazole (400 mg/day) in a case with amphotericin B MIC = 2 μg/mL.^[9]^ In our amphotericin B-resistant case (MIC = 2 mg/L), treatment failure occurred with both induction regimens (AmB-D/fluconazole for 14 days, followed by fluconazole/flucytosine for 29 days). Notably, a clinical response was achieved through salvage therapy combining ABCD and flucytosine, followed by consolidation therapy.

### 3.1. Why does AmBd fail to treat amphotericin B-resistant CM, whereas ABCD is effective?

#### 3.1.1. Amphotericin B resistance mechanisms and therapeutic challenges

The development of amphotericin B resistance in *C neoformans* involves multiple pathways: PKH3-mediated resistance mechanisms that alter membrane ergosterol composition^[6]^ and biofilm formation and virulence factor-induced drug tolerance.^[1]^ Despite these resistance patterns, therapeutic strategies such as dose escalation or combination antifungal therapy may overcome microbial tolerance.^[10]^ In the present case, the initial treatment failure with the AmB-D/fluconazole combination may be attributed to 3 key factors: suboptimal CSF penetration: the maximum tolerated dose of AmB-D in Chinese patients (0.6 mg/kg/day) results in inadequate cerebrospinal fluid concentrations; delayed therapeutic levels: gradual dose escalation protocol prevented rapid achievement of fungicidal concentrations; pharmacodynamic antagonism: concurrent administration led to drug interactions when both agents reach the infection sites within 2 to 4 hours.^[11]^

#### 3.1.2. Therapeutic advantages of lipid formulations of amphotericin B formulations

Given the pharmacological limitations of AmB-D, substitution with lipid-based formulations represents a significant therapeutic advancement. Current evidence demonstrates that liposomal amphotericin B provides 3 key clinical benefits^[4,12,13]^: enhanced dosing flexibility (up to 10 mg/kg/day), tolerance of rapid infusion rates (≤120 min), and superior blood–brain barrier penetration, collectively enabling faster fungal clearance and improved clinical responses in CM.

ABCD, China’s first generic lipid complex formulation (approved March 2021), exhibits comparable CSF pharmacokinetics to liposomal amphotericin B while offering distinct pharmacological advantages.^[14]^ Its unique cholesterol complex structure enables: selective fungal ergosterol targeting, minimized host cell membrane binding, and overcoming microenvironment-induced phenotypic resistance. Emerging Chinese real-world data also demonstrate ABCD’s clinical utility of ABCD in amphotericin B-resistant CM and lower nephrotoxicity.^[15,16]^ While clinical experience with ABCD for CM remains limited, our case demonstrates its efficacy as salvage therapy when combined with flucytosine.These findings highlight the need for: expanded clinical trials in CM populations; pharmacoeconomic evaluations in resource-limited settings; standardized therapeutic drug-monitoring protocols.

### 3.2. The critical role of pharmacist intervention in cryptococcal meningitis management

CM necessitates extended, high-intensity antifungal therapy, where treatment failure frequently results from adverse drug reaction (ADR)-related discontinuations. As the therapeutic cornerstone for CM, amphotericin B is associated with 3 major ADR profiles^[16]^: nephrotoxicity (affecting 30–50% of recipients), hematologic toxicities (dose-dependent decrease), and infusion reactions (mediated through TLR2/CD14-dependent proinflammatory cytokine release). Notably, ABCD demonstrates more severe infusion-related toxicity than conventional AmB-D.^[17]^ Current guidelines recommend^[16]^ a protocol with dexamethasone 1 to 5 mg IV 20 to 30 minutes pre-infusion. Generally, short-term is used for prophylaxis, as reaction severity diminishes with repeated exposure to ABCD.^[16,18,19]^ This case revealed 3 critical pharmaceutical care deficiencies: unmonitored dexamethasone use: prolonged high-dose dexamethasone without toxicity surveillance; risk communication failure: inadequate physician education regarding ABCD reaction profiles; preventable complication: fatal multidrug-resistant K pneumoniae meningitis secondary to iatrogenic immunosuppression.

These findings underscore the importance of systematic pharmaceutical interventions (including therapeutic drug monitoring, multidisciplinary risk communication, and protocolized dexamethasone tapering) are essential for optimizing CM treatment adherence and mitigating ADR-related morbidity.

## 4. Conclusion

ABCD is a feasible alternative for amphotericin B-resistant *C neoformans* meningitis, particularly in resource-limited settings or in renally impaired patients, supported by Chinese real-world evidence showing non-inferior CSF penetration and high salvage efficacy. As this is a single case report with limited ABCD-CM data, broader validation is warranted prior to clinical adoption. During prolonged ABCD therapy, pharmacists must implement pharmaceutical care to ensure medication safety and mitigate adverse effects, thereby preventing treatment discontinuation or failure.

## Author contributions

**Conceptualization:** Liang Long.

**Data curation:** Yi Yan.

**Methodology:** Can Xiao.

**Supervision:** Xiang Liu.

**Visualization:** Qingzi Yan.

**Writing – original draft:** Liang Long.

**Writing – review & editing:** Qingzi Yan.
